# Assessment of the prevalence and consistency of microvascular flow imaging patterns in focal nodular hyperplasia

**DOI:** 10.3389/pore.2026.1612253

**Published:** 2026-01-23

**Authors:** Boglárka Zsély, Aladár Dávid Rónaszéki, Marco Himsel, Zita Zsombor, Gabriella Győri, Anikó Folhoffer, Dorottya Mühl, Viktor Bérczi, Byung-So Park, Damján Pekli, Oszkár Hahn, András Kiss, Pál Maurovich-Horvat, Pál Novák Kaposi

**Affiliations:** 1 Medical Imaging Centre, Semmelweis University, Budapest, Hungary; 2 Department of Internal Medicine and Oncology, Semmelweis University, Budapest, Hungary; 3 Samsung Medison Co Ltd., Gangnam-gu, Republic of Korea; 4 Department of Surgery, Transplantation and Gastroenterology, Semmelweis University, Budapest, Hungary; 5 Department of Pathology, Forensic and Insurance Medicine, Semmelweis University, Budapest, Hungary

**Keywords:** doppler ultrasonography, focal nodular hyperplasia, liver, microvascular imaging, ultrasound

## Abstract

**Objectives:**

To identify characteristic vascular features of focal nodular hyperplasia (FNH) on microvascular flow imaging (MVFI) and assess the utility of MVFI in FNH diagnosis.

**Methods:**

This retrospective study included B-mode ultrasound (US) and MVFI scans of 41 FNHs, 21 hepatocellular carcinomas (HCCs), 20 metastases (METs), 10 hepatocellular adenomas (HCAs), and eight hemangiomas (HEMs) from 80 patients. Diagnoses were confirmed by contrast-enhanced imaging or histology. Two independent observers evaluated vascular patterns on MVFI. Interobserver agreement was calculated, and logistic regression models using either B-mode or MVFI features were developed to differentiate FNH from other focal liver lesions (FLLs).

**Results:**

Interobserver agreement for MVFI patterns was substantial (κ = 0.641, p < 0.001). The spoke-wheel pattern (OR = 51.53 and 35.28) and central artery (OR = 4.96 and 1.95) were strongly associated with FNH. However, the spoke-wheel pattern also appeared in subsets of HCAs (20%–30%), HCCs (14%–19%), and METs (5%–15%). Rim vascularity was common but nonspecific. The MVFI-based model (AUC = 0.891, p < 0.001) outperformed the B-mode model (AUC = 0.814) in distinguishing FNH. For lesions ≥3 cm, MVFI accuracy was even higher (AUC = 0.944, p < 0.001).

**Conclusion:**

MVFI enhances the diagnostic confidence of US for FNH, particularly in asymptomatic patients at low risk for malignancy. However, given the potential overlap with certain malignant FLLs, MVFI findings should be interpreted with caution.

## Introduction

Ultrasound (US) scans are routinely performed to evaluate focal liver lesions (FLLs) due to their accessibility, affordability, and the fact that they do not expose patients to ionizing radiation [1]. As a result, US is often the first-line imaging modality for assessing abdominal organs. B-mode US is used for surveillance of FLLs in patients with chronic liver disease [2]. However, its diagnostic accuracy is limited by overlapping imaging features among different FLL types, reliance on the operator’s experience, and the technical capabilities of the US equipment [3]. Compared to contrast-enhanced imaging modalities such as computed tomography (CT) or magnetic resonance imaging (MRI), B-mode US generally provides lower diagnostic accuracy [4].

Many FLLs are asymptomatic and are incidentally detected during abdominal US examinations conducted for unrelated reasons. Although most of these so-called “incidentalomas” are benign, they can present a diagnostic challenge, often prompting further testing that may cause anxiety and increase healthcare costs. The reported prevalence of incidentally detected FLLs can be as high as 15.1%, depending on the population studied [3]. The most commonly diagnosed liver incidentalomas include focal fat changes, cysts, hemangiomas (HEMs), and, less frequently, focal nodular hyperplasia (FNH) and hepatocellular adenomas (HCAs).

FNH represents the second most common benign hepatic lesion after hemangioma. It exhibits a strong female predominance, with a female-to-male ratio of approximately 8:1, and is most frequently diagnosed between the third and fifth decades of life [5]. As FNH is benign, with no risk of malignant transformation, asymptomatic lesions do not require treatment follow-up [5]. Therefore, accurate differentiation of FNH from other FLLs, such as HCA and malignant tumors that may require surgical intervention, is essential. Contrast-enhanced imaging, including contrast-enhanced ultrasound (CEUS) or hepatobiliary contrast-enhanced MRI (HB-MRI), is usually needed for confirming the diagnosis [6, 7].

Microvascular flow imaging (MVFI) is an advanced Doppler US technique that utilizes multidirectional wall filters to suppress low-frequency tissue motion while preserving signals from low-velocity blood flow [8]. Compared to CDI, the MVFI offers improved spatial resolution and greater sensitivity for detecting microvascular flow in small-caliber vessels. Several FLLs exhibit distinctive microvascular patterns on MVFI. Among these, the nodular or spotted-dot flow distributions seen in HEMs and the spoke-wheel pattern characteristic of FNH are the most specific according to the literature [9–12]. Despite these observations, no comprehensive data are currently available regarding the diagnostic reliability of MVFI in differentiating between FLLs.

This study aimed to provide a comprehensive assessment of FLLs using MVFI and to evaluate the interobserver reproducibility of characteristic MVFI patterns in the largest reported cohort of FNH cases. Additionally, we demonstrate that incorporating MVFI can significantly enhance the diagnostic accuracy of B-mode US in distinguishing FNH from other FLLs, potentially streamlining the evaluation of hepatic incidentalomas.

## Materials and methods

### Patient selection

The institutional and regional science and research ethics committee of our university approved this single-center retrospective study. Given the retrospective nature of the study, the ethics committee waived the requirement for written informed consent from participants. However, all patients provided written consent for CEUS, contrast-enhanced (CE) CT, MRI examinations, and liver biopsies.

We retrospectively collected scans from 80 patients with 100 FLLs who underwent MVFI as part of abdominal US between June 2021 and November 2023. MVFI was not universally applied to all FLLs, but rather to selected cases in which the examiner determined it could facilitate a correct differential diagnosis. Eligibility criteria included: age 18 years or older, diagnosis or follow-up of FLLs, availability of MVFI scans of the lesions, and diagnosis confirmed by independent imaging studies or pathology report. Patients were excluded if they declined participation, had non-identifiable liver lesions on B-mode US had decompensated chronic liver disease, or the diagnosis could not be established based on imaging and histology studies.

FLL diagnoses were confirmed by dynamic contrast-enhanced imaging, or biopsy, following international recommendations and institutional guidelines. A summary of lesions by category is provided below: HEMs either had a typical hyperechoic appearance and were stable on follow-up (n = 4), or showed early-phase peripheral globular enhancement with delayed centripetal filling on CE imaging (n = 6). FNHs were confirmed by HB-MRI findings including isointensity or hypointensity on T1-weighted images, isointensity or slight hyperintensity on T2-weighted images, early enhancement, lack of venous washout, and iso- or hyperintensity in the hepatobiliary phase with or without a hypoenhancing central scar (n = 19); by CEUS showing a central feeding artery, centrifugal filling, homogeneous arterial enhancement, and no washout in venous or delayed phases (n = 15); or by biopsy (n = 13). HCAs were diagnosed by HB-MRI showing arterial hyperenhancement, isointensity in the venous phase, and hypointensity in the hepatobiliary phase, in lesions <5 cm (n = 9); or confirmed by biopsy or surgical excision (n = 8). METs were identified either by biopsy/surgical excision (n = 12), or based on the presence of multiple newly detected hypoenhancing lesions on CE-CT or MRI in patients with known malignancy (n = 19). HCCs were diagnosed in the context of chronic liver disease based on classic imaging features (early enhancement and washout in portal venous/delayed phases, with or without an enhancing capsule) (n = 21), or by biopsy (n = 20). Cirrhosis was present in five patients diagnosed with HCC (n = 7). In one cirrhotic patient, a lesion (n = 1) with arterial enhancement was diagnosed as FNH on HB-MRI. Among HCC patients, six (n = 7 lesions) had prior transarterial chemoembolization (TACE), two (n = 3) received thermal ablation, and two (n = 3) underwent systemic chemotherapy. Additionally, twelve patients with metastatic disease (n = 14 lesions) received chemotherapy before the MVFI examination.

Demographic data, medical history, and diagnostic results were collected from electronic medical records. Table 1 summarizes the characteristics of the patient cohort and the distribution of lesion types.

**TABLE 1:** Characteristics of the patient population stratified by FLL type.

Patient population	All	FNH	HCC	HCA	HEM	MET
Total patients (lesions)^a^	80	31 (41)	18 (21)	6 (10)	8 (8)	17 (20)
Female^a^	45	24 (30)	4 (4)	6 (10)	3 (3)	8 (10)
Male^a^	35	7 (11)	14 (17)	0 (0)	5 (5)	9 (10)
Median age (range)^b^	48 (20–83)	44 (20–68)	70 (56–83)	41 (26–45)	59.5 (36–82)	50.5 (39–80)

Liver status	All	FNH	HCC	HCA	HEM	MET
Steatosis hepatis (lesions) ^a^	18 (26)	9 (14)	2 (2)	1 (4)	4 (4)	2 (2)
Cirrhosis hepatis^a^	11 (14)	1 (1)	5 (7)	0	2 (2)	3 (4)
Treated with chemotherapy ^a^	14 (17)	0	2 (3)	0	0	12 (14)
Treated with embolisation ^a^	6 (7)	0	6 (7)	0	0	0
Treated with thermal ablation^a^	2 (3)	0	2 (3)	0	0	0

FNH: focal nodular hyperplasia, HCA: hepatocellular adenoma, HCC: hepatocellular carcinoma, HEM: hemangioma, MET: metastasis.

^a^ Lesion counts in parentheses.

^b^ Age range in years.

### Microvascular flow imaging of hepatic lesions

All patients were examined using a Samsung RS85 Prestige US system (Samsung Medison Co., Ltd., Seoul, Korea) with a CA1-7S convex probe. Scans were performed after at least 4 h of fasting, with patients positioned supine or in left lateral decubitus, arms raised above the head. All examinations were conducted by a radiologist with over 10 years of experience in abdominal US. Lesions were first identified on B-mode US. The MV-Flow™ mode was then activated to assess microvascular flow patterns. Each lesion was visualized in B-mode, after which the MV-Flow™ window was applied. Still images and 5–10 s video clips were recorded while patients held their breath. Imaging settings were optimized for patient body habitus and lesion location, with typical parameters including an average flow velocity <2 cm/s, dynamic range of 50 dB, and frame rate of 40 fps. The focus was adjusted according to lesion depth. In 27 FNH lesions, vascularity was also evaluated using directional Power Doppler imaging (PDI).

### Multi-reader evaluation of microvascular flow patterns

**FIGURE 1:**
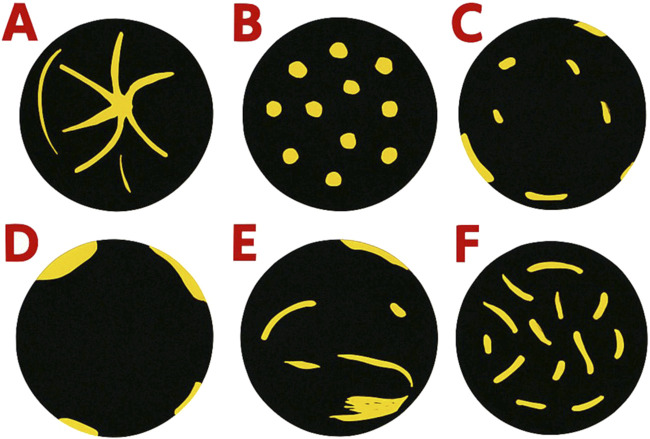
Common microvascular flow patterns observed in the study: **(A)** spoke-wheel pattern, **(B)** spotty dot-like pattern, **(C)** nodular rim with a dot-like pattern, **(D)** hypovascular pattern, **(E)** non-specific vascular pattern, and **(F)** basket-weave pattern.

US and MVFI images were retrospectively anonymized and evaluated. Lesion size, echogenicity, margin clarity, depth from liver surface (DFS), and segmental location were assessed on B-mode images. Two independent consultant radiologists, each with more than 10 years of experience and blinded to clinical data and each other’s findings, reviewed the anonymized MVFI images. Lesions were categorized based on established MVFI flow patterns (Figure 1). Although no standardized classification scheme for evaluating MVFI patterns has been established, previous studies consistently describe a spoke-wheel pattern for FNH; non-specific hypervascular patterns for other primary liver tumors; dotted or nodular rim patterns for hemangiomas; and hypovascular or rim-type vascularity for most secondary liver lesions [9, 10, 13]. The MVFI patterns used to assess vascularity in the present study reflect these principles, are identical to those reported in an earlier publication from our research group, and have demonstrated good discriminatory ability among commonly encountered FLL types [12].

The observers also assessed the presence of a central artery, vascular rim, vascular density, and technical image quality using a three-level scale (poor, acceptable, good). Details of evaluated features are listed in Supplementary Table S1.

### Statistical analysis

Quantitative variables with normal distribution are reported as mean ± standard deviation (SD); non-normally distributed variables are presented as median and range. Categorical variables are shown as frequencies and percentages. The Kruskal–Wallis test was used to compare patient ages across lesion types. Fisher’s exact test assessed the distribution of MVFI features among FLLs. Odds ratios (ORs) and 95% confidence intervals (CIs) were calculated to assess associations between MVFI patterns and lesion types, comparing FNHs to other FLLs. Inter-rater agreement was evaluated using Cohen’s kappa (ƙ). Ordinal logistic regression was applied to identify factors influencing MVFI image quality. Logistic regression models using combinations of MVFI and grayscale features were trained with leave-one-out cross-validation (LOOCV) to distinguish FNH from other FLLs. Diagnostic performance was evaluated using receiver operating characteristic (ROC) curves, and the area under the ROC curve (AUC) was reported.

*A priori* power analysis was performed to ensure a type II error rate below 20% in the ROC analysis [14]. Diagnostic metrics: sensitivity (Se), specificity (Sp), positive predictive value (PPV), and negative predictive value (NPV) were calculated for all lesions, as well as separately for lesions <3 cm and ≥3 cm in diameter. Model comparisons were made using the likelihood ratio test.

Statistical significance was set at p < 0.05. The Dunn-Sidak correction was applied for multiple comparisons [15]. All analyses were performed using R software (version 4.3.2;^1^, accessed 01/12/2023).

## Results

### Characteristics of the patient cohort

The primary objective of this retrospective case–control study was to evaluate MVFI patterns capable of distinguishing FNH from other common FLLs. To achieve this, we aimed to include a substantial number of FNH cases for a comprehensive assessment of their vascular characteristics, along with a control group representing the typical differential diagnoses of FNH, thereby enabling us to assess the diagnostic potential of MVFI. In total, we enrolled 80 patients with 100 FLLs. Because this was a retrospective case–control design focused specifically on comparing FNH with other focal liver lesions, the distribution of lesion types in our sample does not reflect their true incidence in clinical practice. Consequently, FNH constituted the largest subgroup in our cohort (n = 41). It was diagnosed in 31 patients, of which 24 were females with a median age of 44.5 years (range: 20–66 years), and 7 were males with a median age of 40 years (range: 22–68 years). Three patients had three, four patients had two, and twenty-four patients had a single lesion. The second most common FLL was HCC (n = 21), which was diagnosed in 18 patients, including four females with a median age of 77.5 years (range: 65–83 years) and 14 males with a median age of 69 years (range: 56–79 years). HCAs (n = 10) were present in six females with a median age of 41 years (range: 26–45 years). The study cohort also included 17 patients with METs (n = 20) originating from various types of primary tumors, and eight patients diagnosed with HEM (n = 8).

As expected, patients diagnosed with FNH or HCA were significantly younger than those with hepatocellular carcinoma HCC or MET, with all pairwise comparisons yielding p-values <0.012. Male sex was a significant risk factor for HCC, with an OR of 0.12 (95% CI: 0.03–0.35; p < 0.0001), while HCA was observed exclusively in female patients within our cohort.

Hepatic steatosis was identified in 18 patients (22.5%), who collectively harbored 26 FLLs. Background liver cirrhosis was present in 11 patients and was associated with seven HCCs, three METs, two HEMs, and one case of FNH. Prior to MVFI, 22 patients had received locoregional or systemic treatment, involving 13 cases of HCC and 14 METs.

### Evaluation of grayscale ultrasound features

We recorded lesion size, anatomical location, echogenicity, margin definition, DFS for all FLLs. The median size of FNH lesions was 38 mm (range: 12–83 mm), and the median DFS was 34 mm (range: 12–88 mm), with no statistically significant differences compared to other FLL types. FNH lesions were most commonly isoechoic relative to the surrounding liver parenchyma (n = 25; 51%), followed by hypoechoic (n = 11; 27%) and slightly hyperechoic (n = 5; 12%) appearances. The majority of FNHs were located in the right hepatic lobe (n = 25; 61%) and had a peripheral distribution (n = 27; 66%).

Compared to other FLL types, FNH lesions were significantly more likely to appear isoechoic (OR = 11.61; 95% CI: 4.43–33.92; p < 0.0001) and to have poorly defined margins (OR = 4.17; 95% CI: 1.72–10.00; p = 0.0014). A central scar was identified in 4 FNH lesions, accounting for 10% of cases.

### Common MVFI patterns detected in FNH

**FIGURE 2:**
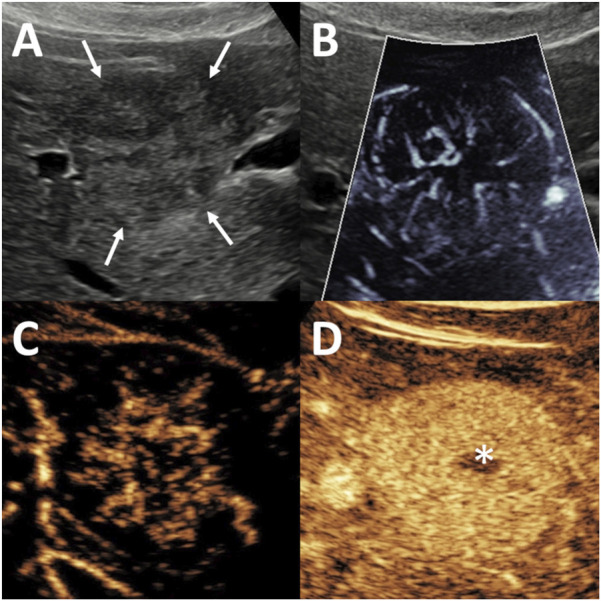
Evaluation of focal nodular hyperplasia (FNH) using microvascular imaging (MVFI) and contrast-enhanced ultrasound (CEUS). The lesion, measuring 20 mm in diameter, was located in segment 5, right beneath the liver capsule. **(A)** B-mode ultrasound (US) shows a nearly isoechoic lesion with poorly defined margins (arrows). **(B)** MVFI reveals a characteristic spoke-wheel vascular pattern. **(C)** In the early arterial phase of CEUS, the lesion demonstrates centrifugal enhancement with stellate vascularity. **(D)** During the venous phase, the lesion remains hyperenhancing relative to the surrounding liver, and a centrally located hypoenhancing scar (asterisk) is visible.

The spoke-wheel vascular pattern was the characteristic MVFI feature of FNH. It was identified in 35 FNH lesions (85%) by the first observer and in 26 lesions (88%) by the second observer (Figure 2). A non-specific hypervascular pattern was observed in 4 FNHs (10%) by the first observer and in 3 FNHs (7%) by the second. Alternative MVFI patterns, including spotted-dot, nodular rim with central dot, and basket-wave configurations, were each observed in only one FNH lesion (2%) by either observer. Among FNH lesions measuring <3 cm, the spoke-wheel pattern was detected in 12 cases (80%) by both observers. The spoke-wheel pattern was highly specific for FNH, as indicated by the observations of the first (OR = 51.53; 95% CI: 16.68–191.51; p < 0.001) and second observer (OR = 35.28; 95% CI: 12.00–124.65; p < 0.001).

However, spoke-wheel-like appearances were occasionally identified in non-FNH lesions: the first observer noted this pattern in 3 HCCs (14%), 2 HCAs (20%), and 1 MET (5%), the second observer in 4 HCCs (19%), 3 HCAs (30%), and 3 METs (15%).

**FIGURE 3:**
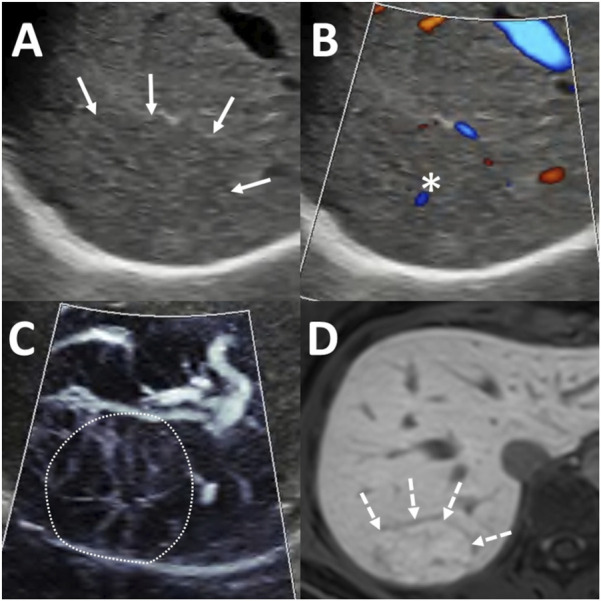
Diagnosis of focal nodular hyperplasia (FNH) using ultrasound (US) and magnetic resonance imaging (MRI). A 65 mm lesion was located beneath the diaphragm in segment 7 **(A)** Greyscale US showed an isoechoic lesion with indistinct margins (arrows). **(B)** Color-coded power Doppler imaging (PDI) highlighted the flow signal in a central artery (asterisk). **(C)** Microvascular imaging (MVFI) revealed a spoke-wheel vascular pattern within the lesion (outlined by the dotted line). **(D)** On hepatobiliary phase MRI, the lesion appeared hyperintense (dashed arrows) relative to the surrounding liver parenchyma, with a hypoenhancing central scar also visible.

The presence of a central artery on MVFI was found to be a characteristic feature of FNH according to the first observer (OR = 4.96; 95% CI: 1.19–23.00; p < 0.03), but this association was not statistically significant for the second observer (OR = 1.85; 95% CI: 0.45–7.10; p = 0.373). The first observer identified a central artery in 30 FNH lesions (73%), as well as in 4 HCCs (19%), 3 METs (15%), and 2 HCAs (20%) (Figure 3). In comparison, the second observer detected a central artery in 32 FNHs (78%), 8 HCCs (38%), 6 METs (30%), and 3 HCAs (30%).

Hypovascular lesions, including those with sparse or no MVFI signal, accounted for 0% (0/41) of FNHs and 44% (26/59) of other FLLs according to the first observer, and 2% (1/41) of FNHs and 8% (5/59) of other FLLs according to the second observer.

### Interobserver reproducibility of MVFI

**FIGURE 4:**
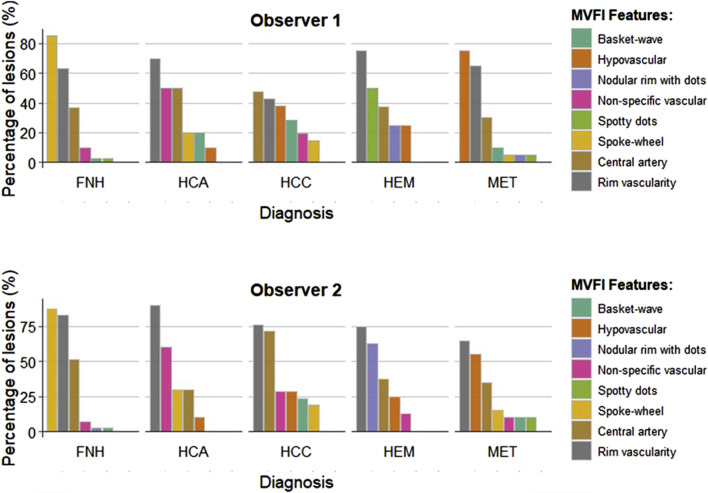
Comparison of MVFI patterns identified by the two observers. MVFI features were independently assessed by the first observer and the second observer across different types of focal liver lesions (FLLs). The color bars represent the percentages of positive lesions within each category. FNH: focal nodular hyperplasia; HCA: hepatocellular adenoma; HCC: hepatocellular carcinoma; HEM: hemangioma; MET: metastasis.

We assessed the interobserver reliability of MVFI features between the two observers. Substantial agreement was observed for the six predefined MVFI vascular patterns (κ = 0.641; p < 0.001) (Figure 4). The reproducibility of the spoke-wheel pattern was even higher, with a κ value of 0.748 (p < 0.001). Agreement was also substantial for the presence of a central artery (κ = 0.638; p < 0.001), while interobserver consistency for rim vascularity was moderate (κ = 0.475; p < 0.001).

### Factors affecting the quality of MVFI images

Image quality was rated by both observers using a three-tier scale. According to the first observer’s evaluation, increasing DFS was significantly associated with poorer image quality (OR = 1.64; 95% confidence interval CI: 1.30–2.11; p < 0.001). Additionally, lesions located in the left hepatic lobe were more likely to receive lower quality scores (OR = 2.00; 95% CI: 1.05–8.52; p = 0.045). In contrast, the second observer did not find a statistically significant association between image quality and DFS (OR = 1.22; 95% CI: 0.98–1.53; p = 0.077) or lesion localization (OR = 0.72; 95% CI: 0.25–1.96; p = 0.552). Lesion size and central versus peripheral location were not significantly associated with image quality in the assessments of either observer.

### MVFI-based diagnosis of FNH

Data from both observers were combined into a single dataset, and logistic regression models were developed using LOOCV to differentiate FNH from other types of FLLs (Table 2). A model incorporating MVFI features, specifically, the presence or absence of a spoke-wheel pattern and central artery, demonstrated significantly higher diagnostic accuracy (AUC = 0.891; p < 0.001) compared to a model based solely on grayscale US features, such as echogenicity and margin definition (AUC = 0.814).

**TABLE 2:** Diagnostic performance of prediction models.

Model^a^	AUC	95% CI	Specificity	Sensitivity	PPV	NPV	Accuracy
MVFI model (spoke-wheel pattern + central artery)							
All lesions	0.891	0.845–0.938	0.864	0.866	0.816	0.903	0.865
≥3 cm lesions	0.944	0.898–0.989	0.917	0.904	0.904	0.917	0.911
Grayscale US model (echogenicity + central scar + margin definition)							
All lesions	0.814	0.752–0.876	0.831	0.756	0.756	0.831	0.8
≥3 cm lesions	0.879	0.818–0.941	0.9	0.808	0.875	0.844	0.857

AUC: area under the receiver operating characteristics curve; CI: confidence interval; MVFI: microvascular flow imaging; NPV: negative predictive value; PPV: positive predictive value; US: ultrasound.

^a^ The metrics were calculated after model selection with leave-one-out cross-validation.

The MVFI-based model misclassified 6 FNH lesions (15%), 3 HCCs (14%), 2 HCAs (20%), and 1 MET (5%) according to the first observer’s evaluation. Based on the second observer’s assessment, the model misclassified 5 FNHs (12%), 4 HCCs (19%), 3 HCAs (30%), and 3 METs (15%) (Supplementary Table S2).

Notably, the MVFI-based model showed even greater diagnostic accuracy (AUC = 0.944, p < 0.001) and consistently outperformed the classic grayscale model (AUC = 0.879) for FNH lesions measuring ≥3 cm.

## Discussion

FNH is a benign lesion characterized by a proliferation of functional hepatocytes and bile duct epithelial cells, lacking normal portal tracts, and organized around a central fibrovascular core with radiating fibrous septa containing a central feeding artery and its branches. The hallmark imaging features of FNH reflect its underlying histologic architecture [5]. On grayscale US, lobules of hyperplastic hepatocytes typically appear isoechoic relative to the surrounding liver parenchyma [16]. As a result, lesion borders are often indistinct and may only be visualized when the lesion displaces adjacent hepatic vessels. An exception occurs in cases of diffuse hepatic steatosis, where FNH may appear relatively hypoechoic due to the increased echogenicity of the surrounding fat-laden parenchyma [7]. In our cohort, the majority of FNH lesions were isoechoic (51%), while approximately a quarter (27%) were hypoechoic. Isoechogenicity (OR = 11.6) and indistinct margins (OR = 4.17) were characteristic B-mode features of FNH. These features may help distinguish FNH from other focal liver lesions, particularly when the background liver is free of marked steatosis or cirrhosis. The fibrovascular core forms a central scar, which can be visualized in 20% of the lesions on grayscale US as a hypoechoic structure in the center of the lesion [17]. Although it is a highly specific feature of FNH, we could identify it in only 10% of the lesions. Nevertheless B-mode US in itself is not sufficient to establish a diagnosis.

FNHs are typically supplied by a hypertrophied feeding artery located centrally, which branches into smaller vessels extending toward the periphery. The detection of vascularity in FNH using CDI has shown variable success, primarily influenced by scanner quality and technical parameter optimization [16]. Previous studies have reported that a central arterial signal, either single (78%) or multiple (18%), was detectable in the majority of FNH lesions on CDI [7]. In a pediatric cohort comprising 9 FNH cases, intralesional vascular signals were observed in 89% of lesions using CDI and in 100% using MVFI [18]. The characteristic spoke-wheel pattern was identified in only 11% of cases with CDI but was appreciable in 67% of cases using MVFI. Another study involving 28 FNHs identified a spoke-wheel pattern in 29% of lesions and a hypervascular dendritic pattern in 64% using CDI [19]. MVFI was also more sensitive than CDI or PDI in detecting central and peripheral vessels in HCCs [20, 21]. In our cohort, among the 27 FNH lesions for which PDI data were available, a central artery was visualized in 59% of cases, a non-specific intralesional vascular signal in 19%, and a spoke-wheel pattern in only 7%. In contrast, on MVFI images, the two observers noted a central artery in 73% and 78% of the FNHs. These results underscore the limitations of conventional Doppler modalities and the superiority of MVFI in delineating the vascular architecture of FNH and other FLLs.

MVFI has been utilized in previous studies to assess the vascularity of various FLLs, with strong correlations reported between specific MVFI vascular patterns and distinct FLL types [11]. According to the literature, hemangiomas are typically associated with spotty dot and nodular rim patterns, while HCCs most frequently exhibit basket wave or non-specific vascular patterns [9, 12, 22]. The spoke-wheel pattern, characteristic of FNH, has been described in several studies; however, these reports generally include fewer than ten cases [10, 18, 23]. To our knowledge, the present study represents the most extensive MVFI-based investigation of FNH to date, comprising 41 lesions. In our cohort, the spoke-wheel pattern emerged as a frequent and highly specific feature of FNH, identified by two independent observers in 85% and 87% of cases, respectively, and observed only rarely in other FLL types (OR: 51.53 and 35.28). Aslan et al. investigated MVFI patterns and the vascularity index in malignant FLLs. Their findings support our observations, as the spoke-wheel pattern was identified in only a minority of cases: one out of sixteen HCCs and three out of sixteen non-HCC primary liver tumors, but only by one observer. This pattern was not observed in any metastatic lesions. These results highlight that, among FLLs, only FNH consistently displays the spoke-wheel pattern [24]. A peripheral vascular rim was also commonly observed in FNH, detected in 46% and 78% of cases by the two observers, but was deemed a non-specific feature. In larger lesions, the vascular rim pattern may reflect the displacement of hepatic vessels by the expanding mass. It is also well established that a draining branch of the hepatic vein can occasionally be seen on CEUS or MVFI in cases of FNH [25]. Combining B-mode US with MVFI has improved diagnostic performance, increasing AUC from 0.867 to 0.945 in distinguishing benign from malignant FLLs [26]. In our study, a regression model using MVFI-derived features outperformed a model based solely on greyscale features in diagnosing FNH, achieving higher sensitivity (86.6% vs. 75.6%). This improvement was particularly notable for lesions ≥3 cm. The MVFI-based model misclassified 12% and 15% of all lesions, and 10% and 17% of malignant FLLs, based on assessments by the first and second observers, respectively.

CEUS is a well-established and widely accepted modality for diagnosing FLLs [27]. In a large multicenter study, CEUS demonstrated a diagnostic accuracy of 98.8% and a negative predictive value of 99.2% for identifying FNH [28]. Another multicenter investigation found that lesions smaller than 3.1 cm were more likely to exhibit typical centrifugal enhancement, which resulted in 69.9% diagnostic accuracy. Interestingly, the spoke-wheel pattern was observed in a similar proportion of small (50%) and larger (71%) FNH lesions [7]. Despite its strengths, CEUS is not routinely used as a first-line screening tool. It requires specialized training, limiting its accessibility in general clinical settings. Moreover, the availability of CEUS for routine assessment of FLLs varies by geographic regions and countries. Another major drawback of CEUS is that it is impractical for the characterization of multiple liver lesions [29]. In contrast, MVFI can be easily integrated into the initial greyscale ultrasound examination. It provides real-time, detailed visualization of a lesion’s vascular architecture, which can support the differential diagnosis. To our knowledge, this is among the first investigations to demonstrate that MVFI has sensitivity comparable to that of CEUS for detecting central arterioles and the spoke-wheel vascular architecture in FNH.

The interobserver agreement for identifying MVFI patterns was substantial (κ = 0.641), with even higher agreement observed for the detection of the spoke-wheel pattern (κ = 0.748). These findings suggest that pattern-based interpretation of MVFI scans is a reliable approach. The use of cine images, rather than single-frame images, could have artificially inflated interobserver reproducibility. At the same time, a separate independent study reported similarly strong interobserver agreement among readers evaluating MVFI patterns in malignant FLLs, with an overall kappa value of 0.634, supporting the good interpretability of these imaging patterns [24]. However, the quality of MVFI images was influenced by the depth and location of the lesions. Specifically, image quality decreased with increasing depth due to weakening of the Doppler signals. Additionally, lesions in the left lobe were more prone to image degradation caused by motion artifacts from the proximity of the pulsating heart. These factors represent the most significant limitations of MVFI in clinical practice.

Our study has several limitations: First, it was a single-center, retrospective case-control study with a relatively small number of lesions. Therefore, the findings should be validated in larger, prospective, multicenter cohorts to confirm their generalizability. Second, all examinations were performed using the same US system and MVFI software. Consequently, our results may not be directly applicable to MVFI solutions from other vendors, since technical variations could affect both the sensitivity and interobserver reliability of the method. Third some patients with malignant FLLs had received locoregional or systemic therapy before their inclusion in the study. These treatments may have altered the vascular characteristics of the lesions, potentially influencing their appearance on MVFI. Currently, there is limited data on post-treatment MVFI patterns in FLLs. However, it is anticipated that vascular density will decrease in FLLs following thermal ablation or TACE, a trend well documented with CEUS [30, 31]. One study found that MVFI demonstrated greater sensitivity than CDI in detecting intralesional hypervascularity, which is considered an indicator of residual or recurrent HCC following TACE [32]. Meanwhile, previous studies have also shown that the spoke-wheel pattern is highly specific to FNH, as it is rarely detected in primary liver cancers and completely absent in secondary liver lesions [24]. Therefore, it is unlikely that our analysis was significantly affected in its ability to distinguish FNH from other types of FLLs. It is also important to note that the primary clinical role of MVFI is to support the diagnosis of suspected FNH in asymptomatic patients with a low risk of malignancy. Accordingly, our findings are most relevant to this diagnostic context. Fourth, during ultrasound scans, not all FLLs were assessed with MVFI; only a subset was, for which the examiner deemed it helpful in establishing a correct differential diagnosis. Thus, our patient cohort may not represent the true proportions of FLL types encountered in routine clinical practice, and the analysis may be subject to preselection bias.

## Conclusion

In this study, we evaluated MVFI features in a collection of FNHs and a comparable number of other FLLs. Our findings demonstrate that pattern-based interpretation of MVFI images is reproducible, and that the spoke-wheel pattern, central artery, and peripheral rim vascularity are characteristic features of FNH. MVFI features significantly improved the diagnostic accuracy of non-enhanced US, achieving higher sensitivity and a substantial negative predictive value for FNH. However, the spoke-wheel pattern was also observed in a subset of HCCs, METs, and HCAs. These findings suggest that MVFI is most helpful for confirming FNH in asymptomatic patients with a low risk of malignancy. In such cases, MVFI can provide additional diagnostic confidence when used alongside B-mode imaging and clinical information. Thus, MVFI could be helpful in triaging focal liver lesions by ruling out diagnoses other than FNH, which could streamline subsequent diagnostic work-up and lessen patient anxiety. However, caution is warranted when interpreting MVFI features in patients with suspected malignancy or underlying liver disease, where overlapping vascular patterns may limit its specificity.

## Data Availability

The original contributions presented in the study are included in the article/Supplementary Material, further inquiries can be directed to the corresponding author.

## Footnotes

1
http://www.r-project.org/
