# Supplementary material for: Assessment of the prevalence and consistency of microvascular flow imaging patterns in focal nodular hyperplasia
Source: Pathol Oncol Res. 2026 Jan 23;32:1612253. doi: 10.3389/pore.2026.1612253 (PMC12875999; doi:10.3389/pore.2026.1612253)
Supplement: Supplementary file 1 [file Table1.pdf]

Supplementary table 1: MVI features scored independently by two radiologists

MVI pattern	*Spoke-wheel*<br>*Spotty dot-like*<br>*Nodular rim with a dot-like*<br>*Hypovascular center with marginal vessels*<br>*Non-specific hypervascular*<br>*Basket-weave*
Prediction of the entity	*Malignant*<br>*Benign*
Prediction of the diagnosis	*FNH*<br>*HCC*<br>*MET*<br>*HEM*<br>*HCA*
Vascularity	*Hypervascular*<br>*Isovascular*<br>*Hypovascular*
Central Artery	*Visible*<br>*Not visible*
Rim vascularity	*Visible*<br>*Not visible*
Image quality	*Good*<br>*Acceptable*<br>*Poor*
