# Supplementary material for: Assessment of the prevalence and consistency of microvascular flow imaging patterns in focal nodular hyperplasia
Source: Pathol Oncol Res. 2026 Jan 23;32:1612253. doi: 10.3389/pore.2026.1612253 (PMC12875999; doi:10.3389/pore.2026.1612253)
Supplement: Supplementary file 2 [file Table2.pdf]

Supplementary table 2: Predictions made by the logistic models

MVI model (spoke-wheel patter + central artery)		
	Observer 1*	
DIAGNOSIS	PREDICTED TYPE	
	OTHER	FNH
FNH	6 (15%)	35 (85%)
HCC	18 (86%)	3 (14%)
MET	19 (95%)	1 (5%)
HEM	8 (100%)	0 (0%)
HCA	8 (80%)	2 (20%)
	Observer 2*	
DIAGNOSIS	PREDICTED TYPE	
	OTHER	FNH
FNH	5 (12%)	36 (88%)
HCC	17 (81%)	4 (19%)
MET	17 (85%)	3 (15%)
HEM	8 (100%)	0 (0%)
HCA	7 (70%)	3 (30%)
Greyscale US model (echogenicity + central scar + margin definition)		
DIAGNOSIS	PREDICTED TYPE	
	OTHER	FNH

FNH	13 (32%)	28 (68%)
HCC	17 (80%)	4 (20%)
MET	18 (90%)	2 (10%)
HEM	8 (100%)	0 (0%)
HCA	9 (90%)	1 (10%)

FNH: focal nodular hyperplasia; HCA: hepatocellular adenoma; HCC: hepatocellular carcinoma; HEM: hemangioma; MET: metastasis.

*Predictions were based on the evaluations by either of the observers.
